# Prognosis of undiagnosed chest pain: linked electronic health record cohort study

**DOI:** 10.1136/bmj.j1194

**Published:** 2017-04-03

**Authors:** Kelvin P Jordan, Adam Timmis, Peter Croft, Danielle A van der Windt, Spiros Denaxas, Arturo González-Izquierdo, Richard A Hayward, Pablo Perel, Harry Hemingway

**Affiliations:** 1 Arthritis Research UK Primary Care Centre, Research Institute for Primary Care and Health Sciences, Keele University, Keele ST5 5BG, UK; 2 NIHR Cardiovascular Biomedical Research Unit, Barts Heart Centre, London, UK; 3 Farr Institute of Health Informatics Research London, Institute of Health Informatics, University College London, London, UK; 4 Centre for Global Non-Communicable Diseases, London School of Hygiene and Tropical Medicine, London, UK

## Abstract

**Objective** To ascertain long term cardiovascular outcomes in patients whose chest pain remained undiagnosed six months after first presentation.

**Design** Cohort study.

**Setting** UK electronic health record database (CALIBER) linking primary care, secondary care, coronary registry, and death registry information.

**Participants** 172 180 adults aged ≥18 from 223 general practices presenting with a first episode of recorded chest pain, classified from medical records as diagnosed (non-coronary condition or angina) or undiagnosed (cause unattributed) at first consultation between 2002 and 2009 and with no previous record of cardiovascular disease.

**Main outcome measures** Fatal or non-fatal cardiovascular events over 5.5 years’ follow-up. Adjustments were made for age, sex, deprivation, body mass index, smoking status, year of index presentation, and previous records of diabetes or hypertension or previous prescriptions for lipid lowering drugs.

**Results** At the index presentation, 72.4% of patients (124 688) did not have a cause attributed for their chest pain; 118 687 (95.2%) of these did not receive any type of cardiovascular diagnosis over the next six months. Only a minority of patients in all three groups (non-coronary 2.0% (769 of 39 232); unattributed 11.7% (14 582 of 124 688); angina 31.5% (2606 of 8260)) had a recorded cardiac diagnostic investigation in the first six months after presentation. The long term incidence of cardiovascular events was higher in those whose chest pain remained unattributed after six months (5126 of 109 628; 4.7%) compared with patients with an initial diagnosis of non-coronary pain (1073 of 36 097; 3.0%) (adjusted hazard ratios for 0.5-1 year after presentation: 1.95, 95% confidence interval 1.66 to 2.31; for 1-3 years: 1.35, 1.23 to 1.48); for 3-5.5 years: 1.21, 1.08 to 1.37). Owing to the larger number of patients in the unattributed group, there were more excess myocardial infarctions in the long term in this group (214 more than expected based on the rate in the non-coronary group) than in the angina group (132 more than expected). Patients who had cardiac diagnostic investigations in the first six months had a higher long term risk of cardiovascular events, regardless of the initial chest pain label. Incidence of unattributed chest pain and angina decreased between 2002 (124 per 10 000 person years and 13 per 10 000 person years, respectively) and 2009 (107 per 10 000 person years and 5 per 10 000 person years, respectively), but the incidence of chest pain attributed to a non-coronary cause remained stable (37-40 per 10 000 person years). Risk of cardiovascular events did not change over time.

**Conclusions** Most patients with first onset chest pain do not have a diagnosis recorded at presentation or in the subsequent six months, including those who undergo cardiac investigations. These patients have an increased risk of cardiovascular events for at least five years. Efforts to better assess and reduce the cardiovascular risk of such patients are warranted.

## Introduction

Each year 1-2% of adults in the UK attend primary care with chest pain symptoms for the first time.^1^ ^2^ ^3^ The dominant concern is that these patients may have treatable coronary disease. At the first consultation general practitioners may diagnose myocardial infarction, angina, or a non-coronary cause, such as gastro-oesophageal or musculoskeletal disease or anxiety. Most often the GP will record only the symptom at this stage and not attribute it to any specific cause,^1^ ^2^ ^4^ while pursuing investigations in those for whom coronary heart disease is considered a diagnostic possibility.

Cardiovascular disease will be diagnosed in 2-10% of patients in the unattributed group within 12 months, most within 6-12 weeks of that first presentation.^2^ ^4^ Risk factors for future cardiovascular events are more prevalent in this group than among patients without chest pain.^4^ Patients in the general population who report chest pain and those attending specialist chest pain clinics who are told that they do not have a cardiac cause of their chest pain have a higher future incidence of fatal and non-fatal cardiovascular disease than pain-free populations.^5^ ^6^ Thus, the symptom of chest pain seems to be a marker of cardiovascular risk.

Two important questions remain unanswered. Firstly, do patients who still have unattributed chest pain after an initial period to resolve diagnostic uncertainty have an increased risk of long term fatal and non-fatal cardiovascular events compared with patients diagnosed as having non-coronary chest pain? No study has investigated cardiovascular outcomes in patients with chest pain who remain without a diagnosis of cardiovascular disease, after allowing time for further assessment and short term increases in clinical manifestations of underlying disease. Thus, whether the initial GP diagnosis and investigation provides enough information about disease status to warrant long term monitoring, is unclear.

Secondly, has improved access to diagnostic procedures (such as the introduction of rapid access chest pain clinics) and the observed decrease in the population incidence of coronary heart disease^7^ ^8^ led to changes in diagnostic labelling and prognosis of chest pain in primary care? This has not been previously investigated.

We sought to tackle these questions by studying patients with no record of previous cardiovascular disease, who consulted and were recorded with first onset of chest pain in UK primary care but were not given an immediate diagnosis of myocardial infarction. We used primary care electronic health records linked to hospital, disease registry, and cause specific mortality records to obtain a more complete and accurate evaluation of major cardiovascular disease endpoints.

## Methods

### Setting

The study was set in the CALIBER (cardiovascular disease research using linked bespoke studies and electronic health records) research programme. CALIBER links primary care data from the Clinical Practice Research Datalink (CPRD) to the national registry of acute coronary syndromes (Myocardial Ischaemia National Audit Project, MINAP), inpatient diagnoses and procedures from Hospital Episode Statistics, and cause specific mortality from the Office for National Statistics.^9^ The CPRD is representative of the UK population in terms of sociodemographic characteristics and overall mortality.^10^ ^11^ ^12^ ^13^ We have previously demonstrated the validity of CALIBER data for a wide range of cardiovascular risk factors across a variety of incident cardiovascular events.^14^ ^15^ ^16^ ^17^ A description of the CALIBER approach and phenotyping algorithms combining Read, ICD-10 (international classification of diseases, 10th revision), and drug and procedure codes to define risk factors and endpoints are available at www.caliberresearch.org.

### Study population

The study population was all patients in the database aged 18 or over with a first (incident) coded record of chest pain (denoted as chest pain with cause unattributed, chest pain attributed to non-coronary cause, or angina) in primary or secondary care between 2002 and 2009. We excluded those with a record of angina or cardiovascular disease before their first recorded presentation of chest pain in that period or with less than two years of up-to-standard quality data in the CPRD at the time of their first chest pain event. The first record of chest pain was defined as the index presentation.

### Definitions of chest pain at index presentation

We identified Read and ICD-10 symptom codes (available from www.keele.ac.uk/mrr) through consensus work to define unattributed chest pain (for example, codes for “chest pain not otherwise specified” or “tight chest pain” without clearly specifying a cause of the pain) and non-coronary chest pain (specific attribution to organ systems other than cardiovascular, such as “chest pain oesophagitis”). Read codes record morbidity in UK primary care;^18^ ICD10 codes record morbidity in secondary care.^19^ Angina was defined by Read or ICD-10 codes for angina or at least two prescriptions for nitrates.

### Alternative explanations for chest pain

We looked for recorded potential alternative explanations for chest pain in the 24 months before index presentation with a prevalence of at least 1% in the study population. These were oesophageal reflux, anxiety, depression, chronic obstructive pulmonary disease, chest infection, asthma, osteoarthritis, spinal pain, and cancer. Morbidities were defined using Read and ICD-10 morbidity codes previously developed in CALIBER or through consensus work at Keele University.

### Baseline cardiovascular risk factors

We assessed baseline covariates (age at index presentation, sex, body mass index (BMI), smoking status, neighbourhood deprivation, prescriptions for lipid lowering drugs, and specific comorbidities) for their association with type of recorded chest pain and as potential confounders for the relation between type of chest pain and future cardiovascular disease. BMI and smoking were defined as the closest recorded value before the index date. Deprivation was based on the English index of multiple deprivation 2007 (IMD)^20^ linked to patient postcode, with patients grouped by fifths. The IMD is a weighted aggregate for the local neighbourhood (mean population 1500) of deprivation across seven domains: income; employment; health deprivation and disability; education, skills, and training; barriers to housing and services; living environment; and crime. We identified prescriptions for lipid lowering drugs in the 24 months before the index date. Diabetes and hypertension were included as known risk factors for cardiovascular disease and identified as recorded diagnoses in primary or secondary care in the 24 months before the index presentation.

### Investigations and interventions after index presentation

We determined the prevalence of cardiac diagnostic investigations (coronary angiography (invasive, computed tomography, magnetic resonance imaging), echocardiography (stress, exercise, and resting), and myocardial perfusion scans) in the first six months after the index date. As a measure of clinical intervention to reduce risk of future cardiovascular disease, we identified prescriptions for lipid lowering, hypertension, and diabetic drugs in the six months after the index presentation up to the first cardiovascular episode or six months, whichever was earlier. Prescriptions for nitrates were not included because they were used to define angina and were regarded as treating a symptom rather than reducing risk.

### Outcomes

We assessed two primary outcomes from index presentation to a maximum follow-up of 5.5 years: incident myocardial infarction (fatal or non-fatal) in all patients and any recorded first fatal or non-fatal cardiovascular event in patients with unattributed or non-coronary chest pain. We identified cardiovascular events in the “diagnostic window” of the first six months after index presentation; the next five years was the period of observation for the long term outcomes, which are the main focus of this paper.

Cardiovascular events were defined as: fatal or non-fatal acute myocardial infarction, angina, coronary heart disease not otherwise specified, heart failure, ventricular arrhythmia, cardiac arrest, ischaemic stroke, haemorrhagic stroke, stroke type not specified, transient ischaemic attack, peripheral arterial disease, abdominal aortic aneurysm, sudden cardiac death, percutaneous coronary intervention, and coronary artery bypass graft surgery. We identified these from primary and secondary care records, MINAP, and the ONS death registry, using previously derived algorithms in CALIBER.^9^ We included all cardiovascular events, rather than just those that may initially present with chest pain, as management of people with definite or possible coronary heart disease includes treatments such as statins or hypertension drugs, which may reduce such events (for example, stroke). This is consistent with recommendations to manage cardiovascular disease as a single family of diseases.^21^

### Statistical analysis

We compared the prevalence of baseline cardiovascular risk factors in the three index groups (unattributed chest pain, non-coronary chest pain, and angina) using multinomial logistic regression, further adjusting for year of index presentation and stratifying by age (<65 or ≥65 years). We determined the percentage of patients in each group with a potential alternative explanation for their chest pain.

We compared the incidence of cardiovascular events between groups in two ways. First we derived Kaplan-Meier curves and crude incidence rates from index presentation to 5.5 years. These, therefore, include events during the initial six month diagnostic period as well as long term outcomes.

Our second, and main, analysis determined the incidence of cardiovascular events in those not diagnosed as having cardiovascular disease in the first six months—that is, the long term prognosis from six months to 5.5 years. Analysis in the unattributed and non-coronary groups was restricted to those with no cardiovascular diagnosis or recorded angina in the first six months and with follow-up data that included at least the first six months. We used Cox proportional hazards regression to determine the risk of long term cardiovascular event by type of chest pain at baseline, adjusting for baseline covariates and year of index presentation. Analysis for the outcome of myocardial infarction included those who presented initially with angina but had no other cardiovascular event in the first six months.

The proportional hazards assumption was assessed using Schoenfeld residuals and deemed adequate for the outcome of myocardial infarction. However, for the outcome of any cardiovascular event, the assumption seemed to be violated, so the follow-up time was split into three periods: six months to one year after initial presentation (all patients), one to three years after initial presentation (in those with no cardiovascular event by one year of follow-up), and three to 5.5 years after the index presentation (in those with no cardiovascular event after three years of follow-up). This seemed to give a better model fit than including a (linear) interaction with time or splitting at other time points. Patients were censored by death, end of follow-up period, or end of records in CALIBER.

We repeated the main analysis in the subgroup of patients who had no record of diagnostic investigations within the first six months.

Finally, we calculated the incidence of new presentations of chest pain for each year from 2002 to 2009. The denominator population for each year was the registered population with at least two previous years of up-to-standard quality data. Annual total incidence rates were directly standardised by applying incidence rates to the age-sex general population structure of England in 2009. However, as this made little difference to the incidence figures, we used crude rates to compare across time.

Robust variance estimators were used in all multivariable analyses, accounting for clustering within general practices. We conducted sensitivity analyses for missing data on the baseline covariates. Analysis was performed using SPSS v.21 and Stata/MP 13.0 for Windows.

### Patient involvement

No patients were involved in setting the research question or the outcome measures, nor were they involved in developing plans for design or implementation of the study. No patients were asked to advise on interpretation or writing up of results. There are no plans to disseminate the results of the research to study participants or the relevant patient community.

## Results

The denominator population ranged from 1 158 755 in 2002 to 1 417 211 in 2009. Of these, 172 180 patients from 223 general practices fulfilled the inclusion criteria between 2002 and 2009, with a mean age of 49.0 (SD 17.59). On index presentation, chest pain was recorded as unattributed in 124 688 patients (72.4%), as non-coronary in 39 232 (22.8%), and as angina in 8260 (4.8%). The index presentation was recorded in primary care for 93.5% (160 911) of patients—39 114 in the non-coronary group (99.7%), 115 602 in the unattributed group (92.7%), and 6195 in the group with angina (75.0%). 

### Baseline cardiovascular risk factors

Fewer than 20% of the patients with new recorded unattributed or non-coronary chest pain were aged over 65 (10 258 and 6379 patients, respectively), compared with 58.5% (4828) of patients with new angina (table 1). Being male, having previously recorded hypertension, and obesity were associated with unattributed chest pain and with angina rather than with non-coronary chest pain on the index date (see appendix table 1 in supplementary files online). The percentage of patients with potential alternative explanations for their chest pain in their medical records was generally similar between groups (table 1).

**Table 1 tbl1:** Baseline characteristics

Characteristics	Chest pain (No (%))			Total (n=172 180)
	Non-coronary (n=39 232)	Unattributed (n=124 688)	Angina (n=8260)	
Sex:				
Female	22 625 (57.7)	65 652 (52.7)	4156 (50.3)	92 433 (53.7)
Male	16 607 (42.3)	59 036 (47.3)	4104 (49.7)	79 747 (46.3)
Age:				
18-44	20 159 (51.4)	53 900 (43.2)	563 (6.8)	74 622 (43.3)
45-64	12 694 (32.4)	46 498 (37.3)	2869 (34.7)	62 061 (36.0)
65-74	3588 (9.1)	14 032 (11.3)	2158 (26.1)	19 778 (11.5)
75+	2791 (7.1)	10 258 (8.2)	2670 (32.3)	15 719 (9.1)
Deprivation fifth:				
First (least deprived)	8328 (21.3)	25 698 (20.7)	1568 (19.0)	35 594 (20.7)
Second	7995 (20.5)	25 331 (20.4)	1643 (20.0)	34 969 (20.4)
Third	7664 (19.6)	24 825 (20.0)	1765 (21.4)	34 254 (20.0)
Fourth	7460 (19.1)	24 660 (19.8)	1714 (20.8)	33 834 (19.7)
Fifth (most deprived)	7632 (19.5)	23 738 (19.1)	1545 (18.8)	32 915 (19.2)
Risk factors:				
Diabetes*	1278 (3.3)	5180 (4.2)	1035 (12.5)	7493 (4.4)
Hypertension*	2275 (5.8)	11 348 (9.1)	2526 (30.6)	16 149 (9.4)
Lipid lowering drug*	2304 (5.9)	10 028 (8.0)	2123 (25.7)	14 455 (8.4)
BMI†:				
Normal	14 483 (45.9)	42 074 (41.3)	2114 (30.0)	58 671 (41.7)
Underweight	1006 (3.2)	2529 (2.5)	93 (1.3)	3628 (2.6)
Overweight	10 098 (32.0)	35 261 (34.6)	2888 (41.0)	48 247 (34.3)
Obese	5962 (18.9)	22 114 (21.7)	1947 (27.6)	30 023 (21.4)
Smoking status†:				
Non-smoker	18 557 (54.1)	59 383 (53.8)	3868 (52.8)	81 808 (53.8)
Former smoker	6199 (18.1)	22 373 (20.3)	2096 (28.6)	30 668 (20.2)
Current smoker	9540 (27.8)	28 720 (26.0)	1364 (18.6)	39 624 (26.1)
Serum total cholesterol†:				
≤5	3621 (42.0)	14 273 (39.8)	1721 (40.4)	19 615 (40.2)
>5	5007 (58.0)	21 606 (60.2)	2534 (59.6)	29 147 (59.8)
Alternative explanations*:				
Spinal pain	8346 (21.3)	26 164 (21.0)	1504 (18.2)	36 014 (20.9)
COPD/chest infection	5056 (12.9)	16 026 (12.9)	1264 (15.3)	22 346 (13.0)
Depression	3897 (9.9)	11 780 (9.4)	529 (6.4)	16 206 (9.4)
Gastro-oesophageal reflux	1964 (5.0)	7162 (5.7)	445 (5.4)	9571 (5.6)
Osteoarthritis	1527 (3.9)	5260 (4.2)	694 (8.4)	7481 (4.3)
Anxiety	1643 (4.2)	5475 (4.4)	243 (2.9)	7361 (4.3)
Asthma	1358 (3.5)	4512 (3.6)	243 (2.9)	6113 (3.6)
Cancer	942 (2.4)	3432 (2.8)	422 (5.1)	4796 (2.8)

BMI=body mass index; COPD=chronic obstructive pulmonary disease.

*In the two years before index presentation

†Nearest measurement before index presentation for 81.6% of total population for BMI (140 569/172 180), 88.3% for smoking 152 100/172 180 (88%), total cholesterol serum 48 762/172 180 (28.3%).

### Fatal and non-fatal myocardial infarction and any cardiovascular event during follow-up

The median length of follow-up in patients initially recorded with unattributed or non-coronary chest pain (n=163 920) was 3.3 years (interquartile range 1.6-5.4). At least six months’ follow-up was available for 151 317 (92.3%) patients, and 40 010 (24.4%) patients had the full 5.5 years of follow-up. Kaplan-Meier curves (fig 1) show higher rates of cardiovascular events in the initial six month period than after the six month period and in those with unattributed compared with non-coronary chest pain across the full 5.5 years of follow-up. 

**Figure f1:**
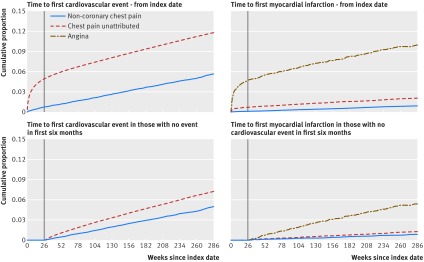
**Fig 1** Kaplan-Meier curves for first cardiovascular event and first myocardial infarction by type of index chest pain

Median times to first cardiovascular event were 135 days (interquartile range 22-664) for patients with unattributed chest pain and 665 (248-1165) days for those with non-coronary chest pain. Rates of cardiovascular events were higher in the unattributed group (292 per 10 000 person years) than in those in the non-coronary group (107 per 10 000 person years) (see appendix table 2). Patients with unattributed chest pain also had a higher rate of myocardial infarction (46 per 10 000 person years) than those with non-coronary chest pain (17 per 10 000 person years) but a lower rate than for those initially recorded with angina (244 per 10 000 person years). Because of the higher number of patients with initially unattributed chest pain, two thirds of myocardial infarctions were in this group (1850 of 2726, 67.9%). Risk of cardiovascular disease was not statistically significantly different by year of index presentation.

### Short term follow-up

In the first six months after the index date, 2.0% (769) of those recorded with non-coronary chest pain (39 232) and 11.7% (14 582) of those with unattributed chest pain (124 668) had a diagnostic investigation (fig 2), compared with 31.5% (2606) of those recorded with angina (8260). Cardiovascular events were recorded in 4.8% (6001) of those with unattributed chest pain and 0.7% (268) of those with initial non-coronary chest pain. Myocardial infarction was recorded in 4.6% (383) of those with initially recorded angina, 0.7% (862) of those with unattributed chest pain, and 0.1% (39) of those with non-coronary pain.

### Long term outcomes

Of the 115 014 patients with unattributed chest pain who were followed up for at least six months, 109 628 (95.3%) remained without a cardiovascular diagnosis at six months (fig 2, table 2). Myocardial infarction rate from 0.5 to 5.5 years was highest in the angina group (hazard ratio 2.56, 95% confidence interval 2.04 to 3.21) and was higher for the unattributed group (1.36, 1.16 to 1.60) compared with the non-coronary group. Owing to the large number of people in the unattributed group, more excess myocardial infarctions occurred from 0.5 to 5.5 years in the unattributed group than in the angina group (214 *v* 132), when comparing age and sex specific rates of myocardial infarctions to the non-coronary group.

**Figure f2:**
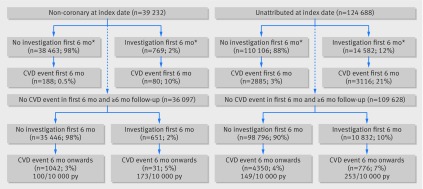
**Fig 2** Cardiovascular events in non-coronary and unattributed groups, stratified by investigations in first six months. CVD=cardiovascular event; py=person years.*Before any cardiovascular event

**Table 2 tbl2:** Association between index chest pain and cardiovascular events in long term follow-up

Type of chest pain at index date	No at risk	Cardiovascular event					Myocardial infarction		
		No (%) with event	Rate per 10 000 person years (95% CI)	Hazard ratio* (95% CI)			No (%) with event	Rate per 10 000 person years (95% CI)	Hazard ratio* (95% CI) 0.5-5.5 years
				0.5-1 year	1-3 years	3-5.5 years			
All:									
Non-coronary	36 097	1073 (3.0)	101 (95 to 107)	1.00	1.00	1.00	174 (0.5)	16 (14 to 19)	1.00
Unattributed	109 628	5126 (4.7)	159 (154 to 163)	1.95 (1.66 to 2.31)	1.35 (1.23 to 1.48)	1.21 (1.08 to 1.37)	829 (0.8)	25 (23 to 27)	1.36 (1.16 to 1.60)
Angina	5573	N/A	N/A	N/A	N/A	N/A	211 (3.8)	116 (101 to 133)	2.56 (2.04 to 3.21)
Subgroup with no diagnostic investigation† in first six months:									
Non-coronary	35 446	1042 (2.9)	100 (94 to 106)	1.00	1.00	1.00	171 (0.5)	16 (14 to 19)	1.00
Unattributed	98 796	4350 (4.4)	149 (145 to 153)	1.86 (1.57 to 2.21)	1.29 (1.18 to 1.42)	1.20 (1.06 to 1.36)	730 (0.7)	24 (23 to 26)	1.34 (1.14 to 1.58)
Angina	4189	N/A	N/A	N/A	N/A	N/A	165 (3.9)	121 (103 to 141)	2.59 (2.03 to 3.31)

N/A=not applicable.

Cohort is patients with no recorded cardiovascular event in first six months.

*Cox proportional hazards regression, adjusted for baseline age, sex, deprivation, body mass index, smoking status, index year, diabetes record, hypertension record, and prescription for lipid lowering drugs in two years before index date.

†Coronary angiogram, echocardiogram, myocardial perfusion scan.

Risk of long term cardiovascular disease was significantly higher over the whole long term follow-up period in patients with initially unattributed chest pain, compared with patients who had a diagnosis of non-coronary pain (0.5-1 year: adjusted hazard ratio 1.95, 95% confidence interval 1.66 to 2.31; 3-5.5 years: 1.21, 1.08 to 1.37). Most cardiovascular events took place in the large unattributed group and represented a 43% excess over expected numbers based on the non-coronary group rate.

The risk of cardiovascular disease in the long term was higher in patients with a diagnostic investigation in the first six months than in those without, regardless of their initial diagnosis (253 per 10 000 person years *v* 149 per 10 000 for those in the initially unattributed group). Of those with no diagnostic investigation in the first six months, the unattributed group had a higher long term risk of a cardiovascular event (149 per 10 000 person years) than the non-coronary group (100 per 10 000 (table 2).

### Early intervention after the index consultation

In the six months after the index consultation, and before any cardiovascular event during this period, the proportion of people prescribed lipid lowering, hypertension, or diabetes drugs was 18.3% (7193 of 39 232) and 26.3% (32 812 of 124 688) in the non-coronary and unattributed groups, respectively. These figures were similar in the subgroup without a recorded cardiovascular event in the first six months (18.1% (6543 of 36 097) *v* 24.9% (27 338 of 109 628)) and higher in those without a recorded event who had undergone cardiac investigations (40.1% (261 of 651) *v* 42.1% (4563 of 10832)) (table 3).

**Table 3 tbl3:** Early intervention in first six months after index date*

Interventions	Non-coronary at index date		Unattributed at index date	
	Total	No (%) who received drug in first 6 months	Total	No (%) who received drug in first 6 months
Prescribed lipid lowering, hypertension, or diabetes drugs:				
All	36 097	6543 (18.1)	109 628	27 338 (24.9)
No investigation in first 6 months	35 446	6282 (17.7)	98 796	22 775 (23.1)
Investigation in first 6 months	651	261 (40.1)	10 832	4563 (42.1)
Prescribed lipid lowering drug:				
All	36 097	2126 (5.9)	109 628	10 067 (9.2)
No investigation in first 6 months	35 446	2026 (5.7)	98 796	7962 (8.1)
Investigation in first 6 months	651	100 (15.4)	10 832	2105 (19.4)

*In those with no cardiovascular event in first six months and follow-up of at least six months.

### Incidence of recorded chest pain

Incidence of recorded unattributed chest pain decreased from 124 per 10 000 person years in 2002 to 107 per 10 000 in 2009. Incidence of recorded angina decreased from 13 per 10 000 person years to 5 per 10 000 over the same period (fig 3). Annual incidence of recorded non-coronary chest pain remained relatively stable (37-40 per 10 000). These trends were apparent across both sexes and all age groups (see appendix figure 1). The percentage of all patients with a new record of chest pain who were coded with angina decreased from 7.8% (1594 of 20 478) in 2002 to 3.6% (767 of 21 049) in 2009.

**Figure f3:**
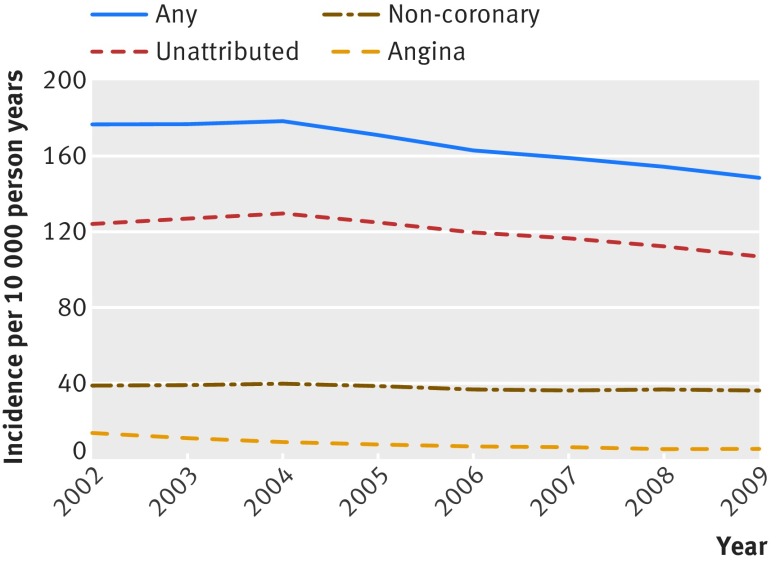
**Fig 3** Trends in incidence of chest pain and angina by year

## Discussion

Most patients presenting with a new episode of chest pain in primary care did not have their pain attributed to a specific cause by the recording GP, and these patients had a higher rate of subsequent cardiovascular events than those whose chest pain was attributed to a non-coronary cause. Six months later most patients with unattributed chest pain had not undergone diagnostic investigations, had not received drugs that might reduce the risk of cardiovascular disease, and had not experienced a cardiovascular event. For the first time, we have shown that patients in this group have a higher long term incidence of any cardiovascular event and of fatal and non-fatal myocardial infarction than those initially diagnosed as having non-coronary chest pain, for up to five years. Rates of myocardial infarction were highest during this period in those who were initially diagnosed as having angina, but the number of patients with myocardial infarction was highest in the group who had remained undiagnosed at six months because of the total number of people in this group. Prognostic models are needed to identify the patients most at risk, so they can be targeted with specific treatment and lifestyle advice directed at reducing the risk of life threatening cardiovascular events.

We found a decline in incidence of unattributed chest pain and of angina from 2002 to 2009. The trend occurred in all age groups, indicating that it was not caused by changes in coding practices or a cohort effect. These patterns reflect declines in the incidence of cardiovascular disease noted elsewhere and are consistent with unattributed chest pain being a disease marker with important prognostic correlates.^7^ ^8^ By contrast the incidence of chest pain attributed to a specific non-coronary diagnosis was stable during this period, indicating that GPs are correctly diagnosing patients at low risk of cardiovascular events. The prognostic relation with cardiovascular outcomes did not change across the index years (2002-09), however, suggesting that diagnostic practice and treatment did not change substantially in these years.

### Comparison with other studies

Our findings are presented in the context of previous evidence about the prognosis of patients with chest pain presenting in primary care.^1^ ^2^ ^3^ ^4^ ^22^ ^23^ They confirm that angina is a minority diagnosis compared with unattributed chest pain.^2^ ^4^ ^24^ They also confirm that a diagnosis of non-coronary chest pain correctly identifies a low risk group.^24^ Our study is novel in investigating what happens subsequently to patients whose chest pain remains undiagnosed in the first six months after presentation, and we have shown that the initial categorisation is related to future myocardial infarction and to any cardiovascular event for up to five years regardless of whether a diagnostic investigation was performed, reflecting the long term prognostic importance of the initial diagnosis.

### Implications

Our findings were robust to multiple adjustments for baseline differences between the three diagnostic groups. Their importance lies in the large number of patients in whom chest pain was unattributed, accounting for more myocardial infarctions during follow-up than patients with a diagnosis of angina, although incidence rates were substantially lower. Implications for clinical practice might include prevention treatment for all patients with unattributed chest pain, even though most will not go on to experience a cardiovascular event. Another option, favoured by Robson et al,^4^ would be to target patients with unattributed chest pain for more detailed prognostic characterisation to identify those at greatest risk more precisely.

GPs will vary in the proportion and type of patients whose chest pain they attribute to specific causes. We did not assess this variability, although our analyses took into account the clustering of patients in practices. Some GPs may intentionally not record an immediate cardiovascular diagnosis, preferring to wait until diagnosis is confirmed. However, this would not affect our main finding of increased long term risk in those still without a diagnosis after six months. The initial classification of patients as having angina or non-coronary disease identified the groups at highest and lowest risk of future events up to 5.5 years. The decision of the GP to do cardiac investigations identified patients more likely to have or to develop cardiovascular problems, even those given a non-coronary diagnosis for their chest pain. Higher proportions of these patients were taking cardioprotective drugs than those not undergoing investigations. 

The “unattributable” label at initial presentation might represent the clinician’s diagnostic uncertainty or their decision to delay formal registration of coronary heart disease until investigations have been completed. Investigations are more likely to be recorded in this group than in the non-coronary group and are more likely to identify a subgroup at risk of developing cardiovascular problems. Another message for clinical practice is that patients with chest pain who are investigated but do not have a cardiac disease diagnosed remain at higher risk of future events than those not referred for investigation, confirming results from an earlier study of referrals to chest pain clinic.^6^ This further supports our conclusion that development of prognostic models could help clinical decision making in primary care to identify which patients with chest pain would benefit from early cardiac investigation and which would benefit from preventive treatments.

Our study shows that initial GP classification of patients with new onset chest pain reflects the likelihood of both underlying current cardiovascular disease and long term risk for future disease. Future research should investigate whether more patients could be placed confidently into the group with initial non-coronary pain, given the low absolute risk of cardiovascular disease in this group. A systematic review of diagnostic indicators of non-cardiovascular chest pain identified several ways in which GP diagnosis of these syndromes might be improved; many patients with unattributed chest pain might be reclassified as definitely having non-coronary chest pain.^25^ Methods to improve diagnosis of coronary heart disease in patients with chest pain would be useful, but clinical algorithms, computerised decision support systems, imaging, and biomarkers have met with variable success in improving the clinical judgment of GPs.^4^ ^26^ ^27^ ^28^ ^29^ ^30^ ^31^ Moreover, the evidence shows that patients “ruled out” of a coronary heart disease diagnosis by these methods remain at increased risk of future cardiovascular events.^6^

An alternate aim of research would be to improve estimation of prognosis in patients with unattributed chest pain and to identify which patients are at highest risk of future cardiovascular disease. Interventions and investigations could then be targeted efficiently and effectively. Such research needs to build on existing healthcare information and risk calculators,^4^ new biomarkers,^32^ and development and validation of prognostic models, as has been done for patients with stable angina.^33^ Improving and testing prognostic estimation in this large group of patients in primary care could, given the excess numbers who progress to preventable cardiovascular events, potentially have substantial impact on population health.

### Strengths and limitations of this study

A strength of our study was the large cohort developed within a national (UK) primary care database with linked hospital, myocardial infarction, and death registry data. Estimates of the annual incidence of chest pain over the eight years of the study (15-18 per 1000 person years) were comparable to those from previous analyses of general practice data in the UK.^1^ ^2^ ^3^ ^4^ The main limitation, in common with other primary care studies based on routinely collected data, is the lack of specific information on the results of diagnostic testing and the possibility that we have missed testing that was recorded only in secondary care. Although some data were missing for some cardiovascular risk factors, analyses using complete cases and analyses using multiple imputation (not shown) delivered similar findings.

### Conclusion

The large group of patients with undiagnosed chest pain in primary care generally do not undergo diagnostic testing but have an increased risk of fatal and non-fatal cardiovascular events for at least five years. More needs to be done to improve the assessment of chest pain in this group and reduce the cardiovascular risk of such patients.

**What is already known on this topic**

Chest pain in primary care is a common diagnostic challenge with potentially serious consequences if cardiovascular disease is missed
Most patients presenting with new chest pain in primary care do not have a specific diagnosis recorded
Several primary care studies show that people presenting with new chest pain and no diagnosis recorded have a higher risk of a cardiovascular event at one year than those with no chest pain

**What this study adds**

Most people who present with unattributed chest pain have no diagnosis recorded over the next six months and do not undergo diagnostic testing
Patients whose chest pain remains unattributed for six months have higher long term risk of major cardiovascular events than those with an initial non-coronary diagnosis for their chest pain
Because of their numbers, patients with unattributed chest pain after six months have the most myocardial infarctions in the long term and should be targeted for better assessment and cardiovascular disease prevention

## Web extra

Extra material supplied by authors

## Notes

Contributors: KPJ, AT, PC, DAvW, PP, and HH designed the study. KPJ wrote the analysis plan and performed the analysis. SD and AG-I prepared the data. KPJ, SD, and RAH defined code lists for exposure, covariates, and outcomes. All authors interpreted the findings. KPJ and PC drafted the paper. All authors contributed to revision of the paper and have approved the final version. KPJ is guarantor. The authors had full access to all of the data in the study and can take responsibility for the integrity of the data and the accuracy of the data analysis.

Ethical approval: This study was approved by the MINAP Academic Group (14-CBR-01) and by the CPRD Independent Scientific Advisory Committee (13_169).

Data sharing: No additional data are available

Transparency: The lead author (KPJ) affirms that this manuscript is an honest, accurate, and transparent account of the study being reported; that no important aspects of the study have been omitted; and that any discrepancies from the study as planned have been explained.
