# Supplementary material for: Prognosis of undiagnosed chest pain: linked electronic health record cohort study
Source: BMJ. 2017 Apr 3;357:j1194. doi: 10.1136/bmj.j1194 (PMC5482346; doi:10.1136/bmj.j1194)

Appendix table 1 - Association of cardiovascular risk factors with type of chest pain on index date, adjusted RRR (95% CI) derived from multinomial logistic regression

		Age < 65		Age ≥ 65	
Chest pain non-coronary is reference group		Chest pain Unattributed	Angina	Chest pain Unattributed	Angina
Female		1.00	1.00	1.00	1.00
Male		1.24 (1.20, 1.28)	1.80 (1.64, 1.96)	1.32 (1.24, 1.41)	1.45 (1.34, 1.57)
Age	18-44	1.00	1.00	-	-
	45-64	1.28 (1.24, 1.33)	5.68 (5.10, 6.32)	-	-
	65-74	-	-	1.00	1.00
	75+	-	-	0.95 (0.90, 1.01)	1.68 (1.55, 1.82)
Deprivation	Least	1.00	1.00	1.00	1.00
quintile	2	1.02 (0.93, 1.11)	1.12 (0.97, 1.29)	1.08 (0.95, 1.23)	1.05 (0.90, 1.24)
	3	1.03 (0.93, 1.14)	1.28 (1.09, 1.51)	1.15 (0.99, 1.34)	1.22 (1.04, 1.43)
	4	1.08 (0.97, 1.22)	1.42 (1.19, 1.68)	1.11 (0.94, 1.30)	1.21 (1.01, 1.46)
	Most	1.03 (0.90, 1.18)	1.42 (1.17, 1.71)	1.16 (0.97, 1.37)	1.28 (1.04, 1.57)
Diabetes[a]	No	1.00	1.00	1.00	1.00
	Yes	0.95 (0.86, 1.05)	1.23 (1.04, 1.45)	0.99 (0.88, 1.10)	1.08 (0.94, 1.24)
Hypertension[a]	No	1.00	1.00	1.00	1.00

	Yes	1.42 (1.31, 1.53)	3.49 (3.08, 3.95)	1.36 (1.26, 1.46)	2.37 (2.17, 2.59)
Lipid-lowering drug prescription[a]	No	1.00	1.00	1.00	1.00
	Yes	1.20 (1.11, 1.29)	2.92 (2.55, 3.34)	1.06 (0.98, 1.15)	1.91 (1.71, 2.13)
BMI[b]	Normal	1.00	1.00	1.00	1.00
	Underweight	0.88 (0.82, 0.96)	0.64 (0.42, 0.98)	1.14 (0.94, 1.39)	0.94 (0.69, 1.29)
	Overweight	1.12 (1.08, 1.15)	1.54 (1.38, 1.71)	1.07 (1.00, 1.14)	1.24 (1.13, 1.36)
	Obese	1.19 (1.14, 1.24)	1.86 (1.66, 2.07)	1.19 (1.10, 1.30)	1.46 (1.29, 1.66)
	Not recorded	1.01 (0.95, 1.07)	1.14 (0.98, 1.32)	1.11 (0.98, 1.26)	1.49 (1.26, 1.76)
Smoking[b]	Non	1.00	1.00	1.00	1.00
	Ex	1.05 (1.00, 1.09)	1.20 (1.08, 1.33)	1.02 (0.95, 1.10)	1.11 (1.00, 1.24)
	Current	0.97 (0.93, 1.00)	1.18 (1.06, 1.31)	0.96 (0.87, 1.06)	0.96 (0.84, 1.11)
	Not recorded	0.90 (0.84, 0.97)	1.00 (0.87, 1.15)	1.04 (0.90, 1.20)	1.21 (1.02, 1.42)

[a] in the two years prior to index date; [b] nearest measurement prior to index date. Multinomial logistic regression, estimates adjusted for presented variables and index year

Appendix Table 2 – Cardiovascular events during full follow-up (0 to 5.5 years) stratified by type of chest pain at index date

	Any cardiovascular event			Myocardial infarction		
Type of chest pain at index date	*n* at risk	*n* with event	Rate per 10,000 person years (95% CI)	*n* at risk	*n* with event	Rate per 10,000 person years (95% CI)
Non-coronary	39,232	1,341	107 (101, 113)	39,232	216	17 (15, 19)
Unattributed	124,668	11,127	292 (287, 298)	124,668	1,850	46 (44, 48)
Angina	N/A	N/A	N/A	8,260	660	244 (226, 264)
Total	163,920	12,468	246 (242, 251)	172,180	2,726	49 (47, 51)

N/A Not applicable

Appendix Figure 1 – Age-gender incidence of chest pain and angina by year

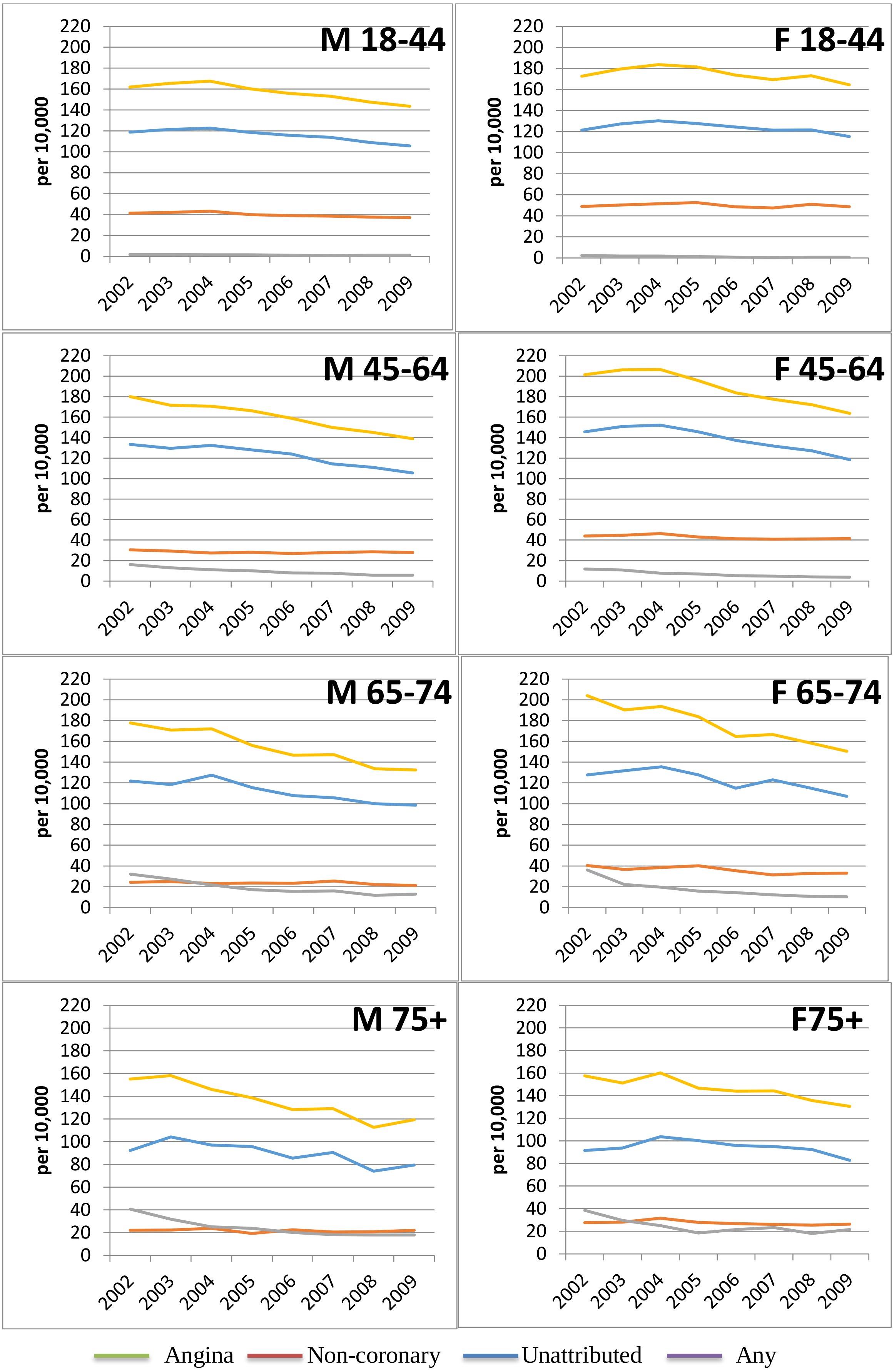

Supplement: Supplementary file 1 — Supplementary information: Supplementary tables and figure [file jork037019.ww1.pdf]
